# Antidotes in Clinical Toxicology—Critical Review

**DOI:** 10.3390/toxics11090723

**Published:** 2023-08-23

**Authors:** Damian Kobylarz, Maciej Noga, Adrian Frydrych, Justyna Milan, Adrian Morawiec, Agata Glaca, Emilia Kucab, Julia Jastrzębska, Karolina Jabłońska, Klaudia Łuc, Gabriela Zdeb, Jakub Pasierb, Joanna Toporowska-Kaźmierak, Szczepan Półchłopek, Paweł Słoma, Magdalena Adamik, Mateusz Banasik, Mateusz Bartoszek, Aleksandra Adamczyk, Patrycja Rędziniak, Paulina Frączkiewicz, Michał Orczyk, Martyna Orzechowska, Paulina Tajchman, Klaudia Dziuba, Rafał Pelczar, Sabina Zima, Yana Nyankovska, Marta Sowińska, Wiktoria Pempuś, Maria Kubacka, Julia Popielska, Patryk Brzezicki, Kamil Jurowski

**Affiliations:** 1 Department of Regulatory and Forensic Toxicology, Institute of Medical Expertises, Łódź, ul. Aleksandrowska 67/93, 91-205 Łódź, Poland; 2 Laboratory of Innovative Toxicological Research and Analyzes, Institute of Medical Studies, Medical College, Rzeszów University, Al. mjr. W. Kopisto 2a, 35-959 Rzeszów, Poland; 3 Toxicological Science Club ‘Paracelsus’, Institute of Medical Studies, Medical College, Rzeszów University, Al. mjr. W. Kopisto 2a, 35-959 Rzeszów, Polandek119600@stud.ur.edu.pl (E.K.); gk091435@stud.ur.edu.pl (G.Z.); mb112731@stud.ur.edu.pl (M.B.); mo112756@stud.ur.edu.pl (M.O.);

**Keywords:** antidotes, toxicology, toxin therapy, critical care

## Abstract

Poisoning and overdose are very important aspects in medicine and toxicology. Chemical weapons pose a threat to civilians, and emergency medicine principles must be followed when dealing with patients who have been poisoned or overdosed. Antidotes have been used for centuries and modern research has led to the development of new antidotes that can accelerate the elimination of toxins from the body. Although some antidotes have become less relevant due to modern intensive care techniques, they can still save lives or reduce the severity of toxicity. The availability of antidotes is crucial, especially in developing countries where intensive care facilities may be limited. This article aims to provide information on specific antidotes, their recommended uses, and potential risks and new uses. In the case of poisoning, supportive therapies are most often used; however, in many cases, the administration of an appropriate antidote saves the patient’s life. In this review, we reviewed the literature on selected antidotes used in the treatment of poisonings. We also characterised the antidotes (bio)chemically. We described the cases in which they are used together with the dosage recommendations. We also analysed the mechanisms of action. In addition, we described alternative methods of using a given substance as a drug, an example of which is *N*-acetylcysteine, which can be used in the treatment of COVID-19. This article was written as part of the implementation of the project of the Polish Ministry of Education and Science, “Toxicovigilance, poisoning prevention, and first aid in poisoning with xenobiotics of current clinical importance in Poland”, grant number SKN/SP/570184/2023.

## 1. Introduction

Antidotes and antivenoms play a crucial role in the treatment of poisoning by specifically targeting the harmful substances involved. Antidotes exert their effects by altering the way the toxic substance behaves in the body, either by affecting its movement or enhancing its elimination. This can also involve interference with the way the toxic substance interacts with receptors, which ultimately leads to better outcomes in cases of poisoning [1].

In the case of poisoning or overdose patients, the first procedure is to follow the principles of emergency medicine, that is, primarily monitoring parameters such as breathing and circulation, as well as supportive care. The American Association of Poison Control Centres Toxic Exposure Surveillance System recommends decontamination, intravenous fluids, and oxygen. In many situations, it is important to administer the correct antidote. Legislation regulates what and in what quantities antidotes should be available in hospitals [2].

Antidotes have been used since the beginning of the history of early medicine and have often been attributed with magical and religious effects. The scientific approach to poisoning therapy with the use of antidotes appeared in the last century and the intensification of research in this area appeared in recent decades due to toxicodynamic and toxicokinetic studies, which contributed to the development of new antidotes. The increased interest in antidotes resulted in the exchange of knowledge and experience on an international scale and the promotion of research in this field. Antidotes are designed to change kinetics and accelerate the elimination of toxins from the body. Currently, they are used in conjunction with other techniques such as gastric lavage, haemodialysis, and hemoperfusion. Some antidotes have become redundant with the advent of modern intensive care. However, in certain circumstances, antidotes can save lives or shorten the duration of toxicity or reduce its severity, increasing the chances of recovery for the poisoned patient. The availability of antidotes is an important concern, especially in developing countries where resuscitation and intensive care facilities are not available [3].

It is also worth mentioning that in recent years there has been a breakthrough in the field of antidotes resulting in the emergence of a new group of supramolecular antidotes. In 2002, sugammadex was developed to reverse the effects of NMBA poisoning. However, its use is still limited due to the risks associated with its use. In addition, other molecules have the potential to become antidotes used in poisoning therapy, among them cyclodextrins, pillararenes, cucurbiturils, and acyclic cucurbituril derivatives should be mentioned. Supramolecular medicine also shows potential in the treatment of other diseases [4].

The objective of this article is to provide information on some of the most important antidotes in medical practise, including their recommended uses and possible risks associated with their use, as well as potential new applications.

## 2. Twenty per Cent or Fat Emulsion (20%) (Lipid Rescue)

Lipid emulsion is an intravenous lipid emulsion used as a dietary supplement for patients who cannot consume enough fat in their diet due to illness or recent surgery. It is prepared from refined safflower or soybean oil and egg yolk phospholipids and contains essential fatty acids such as linoleic acid (LA) and alpha-linolenic acid (ALA) [5].

In addition to being a dietary supplement, lipid emulsions have been found to be effective in treating severe cardiotoxicity caused by intravenous overdose of local anaesthetic drugs such as bupivacaine [6]. They have also emerged as potential rescue therapy for other acute toxicities and poisonings caused by drugs such as tricyclic antidepressants, calcium channel blockers, beta-blockers, antipsychotics, insecticides, and organophosphates.

The mechanism of action of lipid emulsion is not yet fully understood, but several theories have been proposed. The “lipid sink” theory suggests that a lipid compartment is created in the blood into which the lipophilic drug may dissolve, thereby reducing its free concentration in the plasma circulation. Lipid emulsion can also increase cardiac contractility and improve vascular resistance, leading to improved blood pressure and cardiac output [7].

The recommended dosage for lipid emulsion is 1.5 mL/kg (lean body weight) intravenously for 1 min. The bolus can be repeated once or twice in case of persistent cardiovascular collapse. The recommended upper limit is approximately 10–12 mL/kg of fat emulsion in the first 30 min [8].

The side effects of direct fat emulsion infusion are reported in less than 1% of cases and include fever, chills, nausea, vomiting, headache, back pain, chest pain, shortness of breath, and cyanosis [9].

## 3. Acetylcholinesterase Reactivators

The organic compounds called oximes belong to the imines of the general formula RR′C=N-OH, where R is an organic side chain, thus creating a ketoxime, and R may be hydrogen, thus creating an aldoxime. O-substituted oximes form a strictly related family of compounds. Oximes are derived from the condensation of aldehydes or ketones with hydroxylamine (NH_2_OH) [10]. The available reactivators can be structurally classified as charged aldoximes with one or two pyridinium rings connected by different linkers. Hydroxylamine and nicotinohydroxamic acid were shown to be able to reactivate diethylphosphoryl AChE. These initial discoveries quickly led to the discovery that oximes, 2-oxaldoximes, and mainly 2-pyridinium aldoxime (2-PAM) are potent AChE reactivators [11].

Acetylcholinesterase (AChE; EC 3.1.1.7) belongs to the α/β hydrolase family. The main function of AChE is to terminate nerve impulses by hydrolysing the neurotransmitter acetylcholine (ACh) [12]. The biological role of AChE is critical; irreversible inhibition of AChE leads to overstimulation of cholinergic receptors through accumulation of ACh, leading to multisystem failure and finally death [13]. Under physiological conditions, acetylcholine hydrolysis is rapid, which reduces its concentration in neuronal cholinergic synapses and neuromuscular junctions [14]. Irreversible inhibitors include organophosphorus compounds (OP), such as nerve agents (NA) and organophosphorus insecticides [15]. The mechanism of action of nerve agents on the nervous system is through the active site of AChE (Ser–His–Glu triad) [16]. The action is based on the rapid attack of hydroxyl groups in serine, which act as a nucleophile on the NA phosphate groups [11]. Thus, fluoride ions are released, which form a phosphorylated enzyme complex. The effect of creating a covalent bond between phosphorus atoms and AChE serine is to slow (from hours to days) the hydrolysis of acetylcholine [17].

Events such as ageing of the AChE enzyme occur, resulting in its inactivation, which cannot be restored to an active state with any therapy [18]. The aged form of the enzyme is formed as a result of rapid hydrolysis of the =N-O- bond in the OP-AChE adduct, where the phosphonic oxyanion forms a salt bridge with protonated histidine, strongly stabilising the conjugate [19]. Only powerful nucleophiles can restore the functioning of AChE, performed by removing the serine phosphorylating group from the active site of the enzyme. AChE reactivators are considered the most effective antidotes for OP poisoning. Oximes such as pralidoxime, methoxime, trimedoxime, obidoxime, and asoxime are examples of strong nucleophiles [20]. Antidotes capable of restoring AChE activity after intoxication with various NAs (tabun, sarin, and soman of VX) mostly reflect the ternary structural requirements established by these NAs at the active site of AChE. This means that various NA–AChE complexes need a specific oxime to restore their activity [21].

The fine activity of 2-PAM was assigned to the strong binding of the positively charged pyridine to the active site of the enzyme and the correct orientation of the oxime group to displace the phosphoryl moiety. 4-PAM, compared to 2-PAM, was less efficient because its orientation was incorrect. It has been hypothesised that combining 4-PAM with a ligand that is capable of strongly binding to the enzyme could result in a compound with improved affinity and an appropriate orientation of the oxime group at the catalytic site of AChEs [22]. Following this reasoning, TMB-4, the first bispyridine aldoxime, was prepared. TMB-4 turned out to be superior to 2-PAM and 4-PAM due to improved affinity [11]. Then, on the basis of the structure of TMB-4, other aldoximes were synthesised: LüH-6 (Obidoxime), HI-6, and HLö-7 [23]. Although bispyridinium aldoximes are effective reactivators, neither is a universal reactivator. Their reactivation efficiency varies greatly depending on the nature of the phosphine group on the inhibited AChE. Obidoxime is generally considered the best pesticide reactivator [24]. Intravenous obidoxime in a dose of 250 mg reactivates acetylcholinesterase [25]. HI-6 is active against soman and VX but is not effective against tabun. Oximes such as TMB-4, HLö-7, and LüH-6 are effective against tabun inhibition, but their reactivation rate is very slow against VX [26]. Another AChE reactivator is methoxime (MMB-4), a bispyridinium salt. Its synthesis consists of two steps and yields several methoxime salts. Two polymorphic forms of the dichloride and dimesylate salt (MMB4 dichloride and DMS MMB4) were isolated and distinguished using XRD. Both forms (polymorph A and polymorph B) are pharmaceutically acceptable and can be used in the treatment of OP poisoning [27]. Oximes, in addition to their effectiveness as an antidote to nerve gas poisoning, cause side effects such as liver and kidney damage, nausea, and vomiting [20]. The action of reactivators is supported by atropine, which reduces the muscarinic effects caused by nerve agents and pesticides. It acts as a competitive antagonist of central and peripheral muscarinic acetylcholine receptors [25]. Furthermore, Mannich phenol is a non-oxime capable of reactivating the inhibited AChE enzyme [28]. On the basis of the structure of Mannich phenols, several non-oxime compounds capable of reactivating human acetylcholinesterase inhibited by nervous factors were synthesised. This may constitute an effective complementary class of reactivators for the oxime currently used clinically [29].

To reactivate inactive AChE using organophosphorus compounds (OP), antidote pralidoxime (2-PAM) is administered intravenously at a dose of 1–2 g (30 mg/kg bw) for 15–30 min, repeated 60 min later. Intravenous infusions, if administered too quickly, can lead to respiratory or cardiac arrest [30].

Despite the fact that oximes are the only approved AChE reactivators, their therapeutic value is still insufficient due to some limitations. (i) As a result of the variety of OP structures resulting in the formation of different OP–AChE conjugates, none of the known oximes are considered broad-spectrum reactivators. To ensure sufficient reactivation, an oxime that can handle the OP–AChE conjugate and is able to approach and bind the phosphorus atom to the OP is required [31]. (ii) Another disadvantage is related to the high lipophilicity of OP, which results in easy penetration through the BBB (blood–brain barrier), which has a detrimental effect on the central nervous system (CNS). Although OPs readily affect the CNS, common reactivators as charged compounds only slightly penetrate the BBB [32]. The introduction of reactivators with or without charge may facilitate the penetration of the BBB [17]. (iii) Off-target toxicity of oximes is also often discussed as a recent defect [33].

Hundreds of variants have been synthesised and evaluated in recent decades since the discovery of monopyridinium and bispyridine oximes as OP-inhibitory AChE reactivators. All reactivators have three major drawbacks: their constant positive charge prevents them from crossing the blood–brain barrier to reactivate AChE, they show unequal reactivation capacity against AChE inhibited by various types of organophosphorus compounds, and they are ineffective in reactivating “aged” AChE. More research is needed to discover a broad-spectrum reactivator suitable for the entire range of POs. None of the existing pyridinium oximes are true broad-spectrum reactivators [34]. In the short term, a solution to the problem of broad-spectrum reactivators would be to combine two or more oximes that have complementary effects. In this sense, the combination of obidoxim with HI-6 is a promising approach [24]. Further progress in this area will lead to better protection of the public against OPs used in both pest control and warfare.

## 4. Antidotes for Metal Intoxications

### 4.1. BAL

British anti-Lewisite (BAL), or otherwise dimercaprol, 2,3-dimercapto-1-propanol, or 1,2-dithioglycerol, is a heavy metal chelating agent belonging to organic compounds called alkylthiols, compounds containing a functional thiol group attached to an alkyl chain. Its chemical formula is C_3_H_8_OS_2_ and its mean molecular weight is 124.225 [35]. Dimercaprol is a clear, colourless, viscous liquid with a pungent, unpleasant mercaptan odour that boils at 120 °C at 15 mmHg. Its solubility in water is 8.7 g/100 mL and is also soluble in vegetable oils, ether, and ethanol and slightly soluble in chloroform. Its density is 1.2385 at 25 °C/4 °C [36].

The mechanism of action of BAL is that it forms a stable ring, consisting of five members, between the sulfhydryl groups and some of the heavy metals and thus neutralises their toxicity and promotes elimination from the body. After deep intramuscular injection, dimercaprol is rapidly absorbed and its maximum concentration appears 30 to 60 min after injection. Due to its lipophilicity, BAL is distributed in all tissues, concentrating mainly in the liver, kidneys, brain, and small intestine. Due to its short half-life, its degradation and excretion occur in approximately 4 h. BAL is excreted mainly in the urine, but small amounts can also be excreted in the bile [37]. In addition to the treatment of heavy metal poisoning, dimercaprol, because of its copper chelating properties, was originally also used to treat Wilson’s disease, which involves the accumulation of copper in the body. However, due to post-BAL side effects and better agents available for use, it is currently not used for the treatment of Wilson’s disease [38].

BAL was originally developed as an antidote during World War II to counteract Lewisite, a war gas containing arsenic [39]. Today, the drug is used for the treatment of heavy metal poisoning, mainly arsenic, gold, mercury, or lead [40]. BAL is used as a drug in acute poisoning with antimony, arsenic, bismuth, gold, mercury, possibly thallium, and as an adjunct (with sodium calcium edetate) in lead poisoning in children [39]. Some studies suggest that dimercaprol can be used for short-term therapy in patients with severe neurological disorders, as it can penetrate the blood–brain barrier [41]. BAL is administered by deep intramuscular injection into the upper lateral buttocks due to its oily form (it is administered in peanut oil) [37]. The recommended dose of dimercaprol in adult poisoning depends on the metal that the person was poisoned with and its severity [40].

Dimercaprol is administered in combination with sodium calcium edetate and should be administered as first aid at a site different from that of the edetate. In renal disease, BAL can be used normally, but if renal function deteriorates during treatment, therapy should be stopped. In liver failure, the drug is not used, except in arsenic poisoning. Additionally, during dimercaprol therapy, iron preparations should be avoided, as BAL is likely to increase the nephrotoxicity of iron salts. The use of dimercaprol in the treatment of heavy metal poisoning can be associated with numerous side effects depending on the dose used and the time between doses. The most common symptoms are nausea and vomiting, hypertension, and tachycardia. In addition to these symptoms, headaches; weakness; generalised myalgia; lacrimation; salivation; a burning sensation in the eyes, mouth, and nose, sometimes also in the extremities; rhinitis; sweating; or restlessness can occur. Symptoms appear shortly after injection and usually resolve within two hours. Often, haematomas and abscesses or an allergic reaction can also occur at the injection site [40].

Dimercaprol, due to its disadvantages, such as its low therapeutic index, tendency to redistribute heavy metals into the brain or testes, painful injection administration, and unpleasant odour, has now been replaced in the treatment of DMPS and DMSA. These are water-soluble drugs that can be administered orally, so there is no need to prick the patient, and they are associated with fewer side effects. However, they act more slowly and are less effective in removing heavy metals from the body than BAL, implying that dimercaprol may still be used in the treatment of acute heavy metal poisoning [42].

### 4.2. Succimer

Succimer, or meso-2,3-dimercaptosuccinic acid (DMSA), is an oral chelating agent in the treatment of lead and other metal poisoning. It has been used since the 1950s. It is a lipophilic chelator and, therefore, is also a chelating agent for other divalent metals such as mercury, arsenic, cadmium, and gallium arsenide [43]. It is an organic sulphur compound with two sulfhydryl groups with the formula C_4_H_6_O_4_S_2_. Its chemical form is a colourless solid with a molar mass of 182.2 and a melting point of 193 °C, having two carboxyl groups and a stable thiol at room temperature [44]. Succimer is in the form of a white crystalline powder with an unpleasant odour. It contains two mercapto substituents in the 2 and 3 positions. It can be obtained by reacting acetylenedicarboxylic acid with sodium thiosulfate or thioacetic acid, followed by hydrolysis. It is the agent of choice in the treatment of lead poisoning due to its lower toxicity and availability compared to other chelating agents such as Ce-EDTA and dimercaprol (BAL) [45].

Chelation therapy aims to reduce the toxic effects of metals. Currently, hydrophilic dithiol chelators, such as DMPS (2,3-dimercaptopropanesulfonate) and DMSA (meso-2,3-dimercaptosuccinic acid), are used. Chelating agents bind to toxic metal ions, forming a water-soluble complex, allowing them to be easily removed from the body and from extracellular or intracellular spaces. They facilitate the excretion of metals in urine [46]. This is an important parameter due to which it is possible to observe a decrease in the level of lead in the blood and alleviate the symptoms of chronic poisoning with this metal in a measurable way [47]. Its effect as an antidote was first used by Chinese researchers in 1965 [48]. Chelation testing is currently performed in patients with cardiovascular disease and in patients waiting for a kidney transplant who are exposed to lead [49,50]. Recent studies also show that chelation therapy with DMPS and ALA is used to alleviate behavioural deficits, inflammation, and oxidative stress caused by copper [51].

The oral dose for moderate adults is 100 mg three times a day, while in the case of acute poisoning, the dose can be increased by intravenous administration. The elimination half-life is less than 4 h [52]. Scientific studies conducted in 2017 indicate that the use of a dose of 30 mg/kg bw/day for 5 days increased lead excretion in the urine by up to 20 times and blood concentration decreased to 50% [53]. Scientific studies in rats have shown that succimer is fetotoxic at 100 mg/kg bw/day and teratogenic at 400 mg/kg bw/day in mice [54].

A succimer is also used in neuropathy. Scientific studies show that mercury chelation therapy has been tried to treat autism, but this hypothesis was quickly disproved due to the lack of sufficient evidence for excess mercury in people with autism [55].

## 5. Antidotes for Methanol and Ethylene Glycol Poisoning

### 5.1. Ethanol

Ethanol is an alcohol with the chemical formula CH_3_CH_2_OH. It is a colourless liquid with a sharp taste and a characteristic odour. It is miscible with water and has a density of 0.7893 g/cm^3^ [56]. In the 19th century, scientists noticed alcohol’s therapeutic effects. In 1894, their knowledge about ethanol metabolism and its toxicity was summarised with the phrase “It can save and destroy”. Nowadays ethanol is also used as an antidote for methanol or ethylene glycol intoxication [57]. When the scientists noticed that the consumption of ethanol delayed the effects of methanol and ethylene glycol poisoning, they decided that it can be used as a therapy [58].

**Figure 1:**
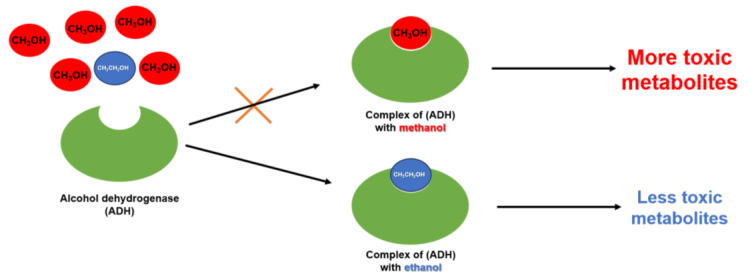
Ethanol prevents methanol from bonding with ADH, the metabolism of which leads to more toxic metabolites.

When a patient has intoxication with ethylene glycol or methanol, doctors can use ethanol as a competitive alcohol dehydrogenase (ADH) substrate to reduce the production of toxic metabolites of methanol and ethylene glycol (Figure 1) [57].

There are different ways in which ethanol can be used as an antidote; the main objective of treatment is to maintain an ethanol level between 1 and 1.5 g/L. This is carried out until the serum concentration of ethylene glycol or methanol has fallen below 0.20 g/L [57]. In this kind of treatment, not only can infusion be used, but ethanol orally prescribed is also effective when intensive monitoring is not available, particularly if the patient is taken to the hospital late [59].

Also, as in every therapy after ethanol treatment, some side effects can be expected. For example, after infusion, a patient may have hypoglycaemia, respiratory depression, hypotension, flushing of gastritis, and others [59].

### 5.2. Fomepizole

Fomepizole (4-methyl-1H-pyrazole) is a derivative of pyrazole with a heterocyclic structure. It is a colourless liquid. In medicine, it is used as an intravenous injection. Fomepizole is an antidote to poisoning with methanol and ethylene glycol [60].

The idea of this antidote is that fomepizole prevents the conversion of ethanol to acetaldehyde and methanol to formaldehyde. This way blocks the next reactions and the formation of the toxic products of their metabolism, i.e., acetic acid and formic acid. It reduces the nephrotoxic effect, protects against optic nerve neuropathy, and the appearance of metabolic acidosis [61].

It should be noted that 4-MP is also used as a prevention for kidney failure caused by ethanol poisoning, in addition to protecting vision and neurological damage after methanol consumption [62]. In the case of poisoning and metabolic acidosis, treatment should be started as soon as possible. The loading dose is 15 mg/kg bw in a slow infusion (30 min). Then, the next doses should be administered; after that we administer the patient 15 mg/kg every 12 h, constantly monitoring the level of ethanol in the blood. Treatment can be stopped when the level of ethanol in the blood is less than 20 mg/dL. It should be underlined that fomepizole is a very useful antidote to ethylene glycol poisoning during pregnancy [63].

Researchers compared the effectiveness of ethanol and fomepizole in the fight against methanol poisoning, and according to the test results, there are practically no differences in clinical effectiveness. Unfortunately, it is likely to have a higher incidence of complications and adverse effects, but it is still used in therapy [57].

### 5.3. Folinic Acid

Folinic acid (5-formyltetrahydrofolate ([R, S]5-CHOFH_4_)) represents more than 90% of functional folate derivatives in plasma, can form crystals, and is sparingly soluble in water [64]. Sometimes, folinic acid is confused with folic acid, but these two terms should not be used interchangeably. 5-formyltetrahydrofolate is a biologically active reduced form of folic acid. It also appears to be a more metabolically active form of folate [65].

Due to the wide range of indications, there are a large number of dosing protocols depending on the situation. Folic acid can be used through various methods of administration, such as intramuscular, intravenous, or oral. The choice of the appropriate time, dose, and route of administration of folinic acid depends on the expected effect in a given medical indication. Typically, folinic acid is administered 24 h after starting methotrexate therapy. It is important to note that the adverse effects of methotrexate may not be reversible unless folinic acid is started approximately 40 h after the administration of methotrexate [66]. Administration of folinic acid allows for the omission of some steps required for folic acid to actively participate in metabolism and gene regulation, because 5-formyltetrahydrofolate does not require dihydrofolate reductase for conversion to tetrahydrofolate [67]. Tetrahydrofolate acts as a single carbon donor in the synthesis of thymidylate and purine. In this way, it participates in the generation of nucleic acids and the regulation of gene expression. Moreover, folinic acid is crucial for the process of synthesis of methionine from homocysteine [68].

Folinic acid is an antidote to the toxic effects of methotrexate and can be used in addition to other pathologies treated with antifolate drugs [69]. Some studies also suggest the possible efficiency of it in treating methanol poisonings [70,71]. Most folic acid antagonists, such as methotrexate, have almost identical schemes of action. Among other things, they inhibit dihydrofolate reductase [72]. As mentioned before, this enzyme plays a crucial role in generating the functional tetrahydrofolate molecule. In clinical practise, folinic acid is generally compounded with calcium in the form of leucovorin calcium. Calcium foliate can be administered intramuscularly, intravenously, or orally. The timing, dosage, and route of administration of folinic acid may vary depending on the desired outcome and indication [67].

## 6. Atropine

Atropine is an organic chemical compound that belongs to the tropane alkaloids group. It is an ester of 3-hydroxytropane and 3-hydroxy-2-phenylpropanoic acid. It comes in the form of white, diamond-shaped crystals that melt at 118 °C. Atropine dissolves in water, but its solubility is best in ethanol. Under laboratory conditions, it exhibits instability when exposed to light. The summary chemical formula of this compound is C_17_H_23_NO_3_ [73].

Atropine is a potent anticholinergic drug that binds and effectively blocks muscarinic cholinergic receptors, thus stopping the stimulating impact of the neurotransmitter ACh. This critical attribute makes atropine, along with other antimuscarinic anticholinergic drugs, a lifesaving necessity for people who face exposure to nerve agents, OP pesticides, or overdoses of other ChE inhibitors. By suppressing secretion and reversing smooth muscle contractions, atropine promptly relieves bronchial spasms, facilitating optimal air exchange and sustaining cardiovascular function. Immediate and forceful administration, sometimes with higher doses of atropine than usual, is imperative to swiftly counteracting the toxic symptoms of nerve agent poisoning in severely affected individuals [74]. Atropine may have a multidirectional effect on the body depending on the target organ [75]:

- Respiratory tract—relaxation of smooth muscles, increasing lumen of the bronchi, reducing mucus secretion.
- Heart—antivagal action on the heart; it increases heart rate and cardiac output and affects the sinoatrial node (to a lesser extent the atrioventricular node), accelerating nodal conduction and shortening the PQ interval.
- Digestive tract—reduces gastrointestinal motility and acts as an antiemetic.
- Valuable for diagnosing sinus node dysfunction, assessing coronary artery disease under atrial pacing conditions, and attempting to restore normal conduction in individuals with Wolff–Parkinson–White syndrome [73,75].
- Premedication before or immediately during the procedure.
- During resuscitation until the heart rhythm is stabilised.
- In bradycardia or arrhythmias.
- Reversed neuromuscular block.
- Auxiliary in spastic conditions of smooth muscle in the abdominal cavity (biliary and renal colic).
- In radiological diagnostics, when it is desired to relax the smooth muscle and slow down the intestinal transit.
- In ophthalmology prior to endoscopy.

It is used in the case of poisoning with organophosphorus compounds, substances with a cholinomimetic effect, or fungi containing muscarine. Atropine can be administered intramuscularly, intravenously, subcutaneously, or intratracheally. In clinical practise, it is used as an antidote by intravenous or intramuscular administration for adults at 1–2 mg, repeated every 5–60 min, if necessary, until symptoms of poisoning are controlled [76].

## 7. Calcium Chloride

Calcium chloride is an ionic compound of calcium and chlorine and behaves as a typical ionic halide. Calcium chloride is produced directly from limestone or as a by-product of the Solvay process [77]. It occurs in monohydrate, dihydrate, tetrahydrate, and hexahydrate forms. It is a white to off-white solid in the form of cubic crystals, granules, or fused masses. The compound is odourless and the taste threshold in potable water is 150–350 ppm. Its boiling and melting points are, respectively, 1935 °C and 782 °C. Calcium chloride is soluble in water, methyl carbonate, acetic acid, and ethanol [78].

Calcium chloride is a compound that has the ability to neutralise or reduce the toxicity effects of overdose β-blockers and calcium channel blockers. In the case of intoxication with beta-blockers, 1000 mg is administered intravenously bolus through the centre line [79].

**Figure 2:**
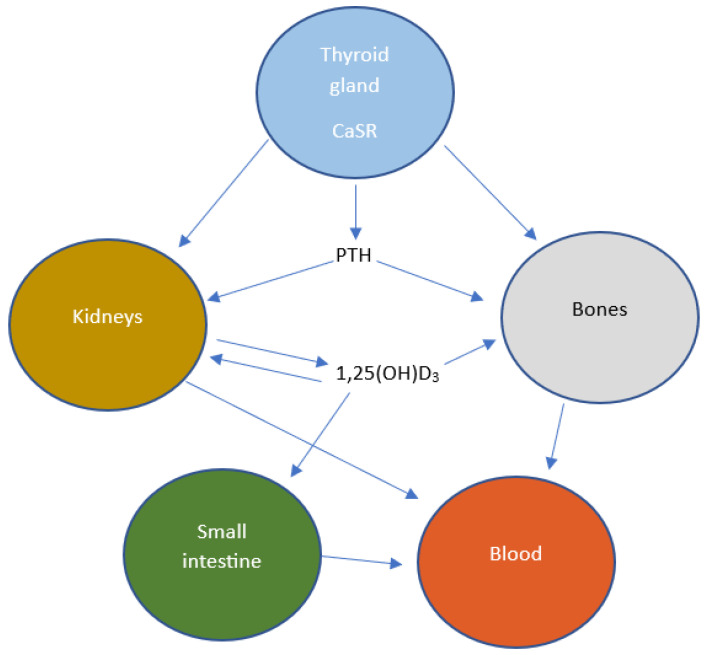
Scheme of calcium homeostasis between bones, blood, small intestine, kidneys and thyroid gland (based on [80]). Parathyroid hormone (PTH) raises blood calcium levels by activating calcium uptake in the kidneys and releasing it from the bones. Additionally, PTH stimulates the production of 1,25-dihydroxyvitamin D3 in kidney cells. This vitamin regulates the absorption of calcium in the intestines, its reabsorption in the kidneys, and its release from the bones.

Calcium chloride is the basic mineral component of the body, which cocreates bone tissue—regulation of calcium concentration levels is crucial to maintaining homeostasis and proper bodily function (Figure 2). It affects the action of numerous enzymes, and is a relay of intracellular information, as well as cell differentiation, immune response, neuronal activity, enzyme activation, and apoptosis. Calcium homeostasis is subject to strict hormonal control—its concentration is increased under the influence of parathyroid hormone and the active form of vitamin D [80].

Calcium chloride is used for highway maintenance: moisture absorbed from the air prevents dust formation; chemical manufacture: production of calcium salts; drying air and gases: direct drying compound; automotive and aluminium: wastewater treatment; mining: dust proofing and freeze-resistant ore and coal; paper manufacture: improves dye retention; petroleum: additive to oil well completion fluids. Calcium chloride is also used to treat acute hypocalcaemia and is indicated for cardiac arrhythmias associated with hypocalcaemia, hypercalcaemia, or hypermagnesemia [79].

## 8. Dantrolene

Dantrolene comprises odourless and tasteless crystals (melting point—279 °C). It has low solubility in water (146 mg/L). The classic preparation of intravenous dantrolene is a lyophilised powder in a 20 mg dose, each with another 3 mg of mannitol that must be dissolved in about 60 mL of sterile water prior to administration [2]. Another formulation is provided in 250 mg vials that require only about 5 mL of sterile water for reconstitution. Such a high concentration of the drug provides a rapid increase in blood concentrations and provides a fast response [81].

**Figure 3:**
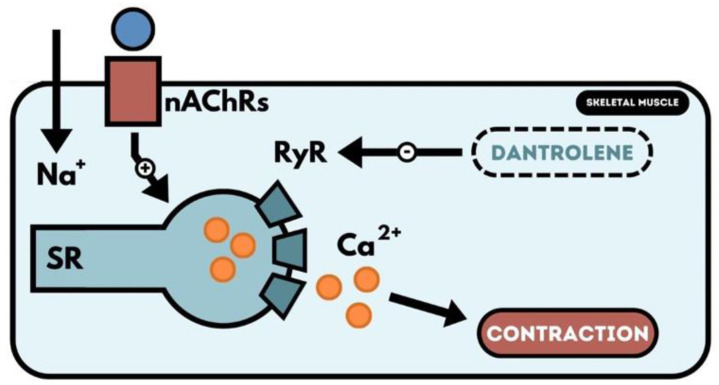
Mechanism of action of dantrolene. Dantrolene is a unique muscle relaxant that targets ryanodine receptors (RYR), which are responsible for intracellular calcium release in muscle cells. By binding to these receptors, it reduces excessive calcium release from the sarcoplasmic reticulum, leading to muscle relaxation and preventing muscle contractions.

The EPP potential generated within the endplate depolarises the sarcolemma of the skeletal muscle, generating an action potential. The action potential spreads along the entire muscle fibre and arrives; it is the transverse intussusception of the sarcolemma, the so-called T tubules. The T tubule membrane is rich in dihydropyridine receptors (DHP-R) that share amino acid sequence homology with the potential-gated ion channel of type CACNA1S. However, the DHP receptor does not function as the ion channel supplying Ca^2+^ ions to the interior cells and serves primarily as the so-called voltage sensor. The action potential reaching the T tubules depolarises the membrane and activates the DHP receptor. This activation causes a channel’s conformation change and shift inside the cytoplasmic cell, a loop that connects the receptor domains II and III. This loop is located a short distance from the RYR ryanodine receptors, which are channels in the smooth endoplasmic reticulum (SR), which stores Ca^2+^ ions. Depolarisation of the T tubule membrane activates the DHP receptor, which in turn opens the ryanodine channel and enters a massive flow of Ca^2+^ ions from the endoplasmic reticulum. There is a high concentration of Ca^2+^ ions within the cell necessary for skeletal muscle contraction (Figure 3) [82].

An increase in the amount of calcium within muscle cells, called myoplasmic calcium, can lead to a cascade of harmful chemical reactions that can cause a condition known as malignant hyperthermia.

Dantrolene is a direct-acting skeletal muscle relaxant that specifically targets muscle cell intracellular calcium release channels called ryanodine receptors (RYR). By binding to these receptors, dantrolene reduces excessive calcium release from the sarcoplasmic reticulum that leads to muscle relaxation by preventing muscle contraction triggering. This drug is unique in its mechanism of action among muscle relaxants, and it is the only clinically approved drug that targets RYR channels.

Dantrolene also affects the ability to obtain normal Mg^2+^ sensitivity of Mg^2+^ to MH-susceptible muscle fibres. Obtaining normal Mg^2+^ sensitivity to the mutated ryanodine receptor may be the next stage of dantrolene therapeusis in MH [83].

Dantrolene is a lifesaving postsynaptic muscle relaxant with a variety of indications in terms of neurology or anaesthesiology. Its main indication is the treatment of malignant hyperthermia, which is a general reaction of our body to depolarising muscle relaxants or anaesthetics, which causes general and persistent muscle contraction. The prevalence of this state is estimated to be 1 in 50,000 to 100,000; however, it is characterised by very high mortality if dantrolene is not administered. Other uses of dantrolene as an antidote include the overdose of 2,4-dinitrophenol—a prohibited medication that causes disrupted ATP synthesis and induced hyperthermia—and the treatment of malignant neuroleptic syndrome.

Dantrolene is used as an intravenous injection or as an oral capsule. The choice of administration method depends on the intended use of the substance. In the treatment of malignant hyperthermia, when symptoms occur, 2.5 mg/kg of dantrolene should immediately be administered intravenously. If symptoms do not alleviate, further doses of 1 to 2.5 mg/kg are indicated up to a maximum cumulative dose of 10 mg/kg. If treatment is successful, additional doses should be administered for another 24 h to prevent recurrence of malignant hyperthermia [81].

Malignant neuroleptic syndrome is another disease in which the use of dantrolene is available. Its symptoms are generally similar to those observed in malignant hyperthermia, including muscle stiffness, mental disorders, and increased body temperature. Administration of dantrolene in malignant neuroleptic syndrome is indicated primarily in the severe stage of the disease—iv dose of 1 to 2.5 mg/kg, and when necessary, increasing the dose to another 1 mg/kg per 6 h up to a maximum daily dose of 10 mg/kg [84].

In addition, there have been other off-label attempts to use dantrolene. One of them is the administration of dantrolene in the treatment of vasospasm induced by aneurysmal subarachnoid haemorrhage. A single dose of iv dantrolene of 2.5 mg/kg was found to decrease the severity of vasospasm. Furthermore, research on Alzheimer’s disease was carried out showning that increased cellular calcium levels are associated with faster progression of the disease and memory loss [85]. Ryanodine receptors are believed to be responsible for increased cellular calcium levels; therefore, the administration of dantrolene in this particular group of patients may serve as a future drug for Alzheimer’s disease, but numerous clinical trials are needed before patients can use dantrolene [81].

Another indication of dantrolene use is chronic muscle spasticity. Among adults, the initial dose of dantrolene is 25 mg orally every day for 7 days; after the first week, the dose may increase and require patient observation before further increase. The maximum dose is up to 400 mg per day [81].

## 9. Diphenhydramine

Diphenhydramine is a 2-(dimethylamino)ethanol benzhydryl ether. Diphenhydramine has a bitter taste and dissolves in the following proportions: 1 g of diphenhydramine dissolves in 1 mL of water, 2 mL of alcohol, 50 mL of acetone, 2 mL of chloroform. It slowly darkens with exposure to light [86].

Diphenhydramine, a first-generation H1 antihistamine, acts on the central nervous system and produces a sedative effect by inhibiting H1 histamine receptors. However, it should be remembered that blocking these receptors is not selective, as diphenhydramine also affects other receptors, mainly cholinergic, and, to a lesser extent, serotonergic receptors and myocardial voltage-dependent sodium channels [87]. It functions as an H1 receptor antagonist and is a cough suppressant. It is a mixture of ether and tertiary amine [86].

Diphenhydramine can be a preventive medicine against the toxic effects of cisplatin on the kidneys. For the treatment of solid tumours, the anticancer drug cisplatin is frequently utilised. Because there is currently no treatment for cisplatin-induced kidney toxicity, this medication has a major side effect restricting its use [88]. The liver metabolises diphenhydramine by CYP450. It is excreted unchanged in urine and has a half-life of 3.4 to 9.2 h. The time to reach the peak of action of the drug in serum is 2 h [89].

H1 receptor antagonist, antiemetic, sedative, antiallergen, muscarinic antagonist, Parkinson’s disease medication, antipruritic medicine, local anaesthetic, antidyskinesia, anticough agent, and oneirogen are some of the additional functions it performs. It is also less frequently utilised in cases of parkinsonism-related tremor and nausea. It can be used topically, injected into a muscle, consumed orally, or injected into a vein [90]. Diphenhydramine reverses the impact of histamine on capillaries by acting as a reverse H1 receptor agonist, reducing allergic reaction symptoms [89]. Muscarinic receptors and the H1 receptor share similarities. Therefore, diphenhydramine is utilised as a treatment for Parkinson’s disease, as it is a competitive muscarinic acetylcholine receptor antagonist that also has antimuscarinic properties. Being a first-generation antihistamine, diphenhydramine rapidly penetrates the blood–brain barrier and inversely antagonises H1 CNS receptors, leading to sleepiness and suppression of the centre of the core cough [91].

Other uses of diphenhydramine are also anticholinergic and sedative. It is one of the medications that Americans (USA) use most frequently. If acute diphenhydramine poisoning is not properly managed right away, it can have catastrophic, even fatal, repercussions [92]. Decontamination methods might be taken into account if the patient reports within one hour after taking diphenhydramine. Since the anticholinergic impact can reduce gastrointestinal motility and result in delayed absorption of diphenhydramine, a single dose of activated carbon may be advantageous. However, unless the respiratory system is protected, the use of charcoal in individuals with altered mental status should be avoided [93]. The effects of diphenhydramine poisoning are currently not specifically treatable with an antidote. Treatment for diphenhydramine toxicity is based on supportive measures that take into account signs and symptoms of poisoning or overdose [94]. There is support for using intravenous therapy for lipid emulsions; nevertheless, its effectiveness in this context is controversial. Haemodialysis may be used in conjunction with diphenhydramine in the treatment of toxicity for this reason [95]. A toxicologist or toxicology centre should be consulted in cases of serious poisoning. The best way to stop reoccurring episodes of unintentional poisoning or overdose is to educate the patient or caregiver [92].

## 10. Flumazenil

Flumazenil (imidazobenzodiazepine) is a white crystalline powder (melting point of 201–203 °C) with solubility in water of 128 mg/L [96]. In medicine, it is used as an injection solution or as a concentrate to prepare a solution for infusion [97].

**Figure 4:**
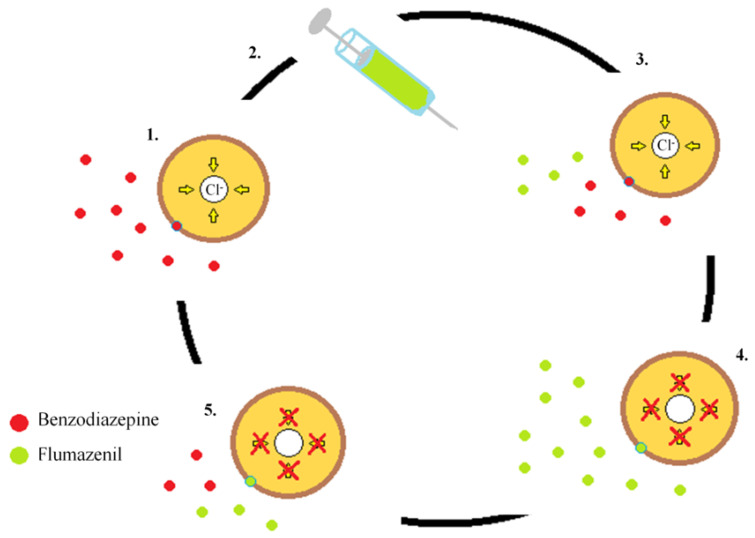
The idea of the action of flumazenil as antidote. 1. Benzodiazepine poisoning—attachment of benzodiazepine to the GABA receptor—centripetal chloride influx. 2. Administration of flumazenil as an antidote. 3. Flumazenil competes with benzodiazepine for the GABA receptor binding site. 4. Attachment of flumazenil to the GABA receptor—reduction of afferent chloride influx. 5. Ending of the flumazenil effect—if the initial dose and concentration of benzodiazepines are high, due to the longer duration of action of benzodiazepines, resedation may occur and the entire cycle is repeated until the desired clinical effect is obtained.

Flumazenil is used as an antidote in the case of an overdose of benzodiazepines, drugs with a sedative, hypnotic, anticonvulsant, and muscle-relaxant effect [98,99]. The mechanism of action of flumazenil is based on interaction with GABA receptors. In the central nervous system, γ-aminobutyric acid (GABA) is the main inhibitory neurotransmitter, and at the same time, the key coordinator of the brain inhibitory effect of GABA is mediated by two types of receptors, GABA_A_ and GABA_B_ [100]. They bind not only to GABA but also to benzodiazepines, whose core structure, including the benzene ring and the diazepine ring, facilitates the binding of γ-aminobutyric acid. The flumazenil binding site is on the extracellular surface of the GABA_A_ receptor at the junction of the α1 i γ2 subunits, in close proximity to the benzodiazepine binding site [101]. Flumazenil acts as a nonspecific competitive agonist on the GABA-benzodiazepine receptor (GABAA), competing with benzodiazepine and rapidly reversing its action by reducing the influx of afferent chloride (Figure 4). The first effects can be noticeable within one minute of flumazenil administration and the maximum therapeutic effect is reached within 6 to 10 min and lasts 19 to 50 min, depending on the initial dose and plasma benzodiazepine concentration [102,103]. After using flumazenil, the patient should be closely monitored because its duration of action is shorter than benzodiazepines and may result in resedation [104].

Overdosing can result both in deliberate action (manipulative self-poisoning—suicide attempts in the form of a “call for help”) and in unintended actions (poisoning related to the development of drug tolerance) [105]. Orally administered flumazenil is easily absorbed, but due to its low hepatic metabolism, it has low bioavailability. Therefore, it is used in intravenous infusions [99,106]. In the case of benzodiazepine poisoning in adults, it results in intensive care. The total dose of flumazenil should not exceed 2 mg in one treatment. In the case of recurrent somnolence, it is possible to use intravenous infusions [98,99]. It should not be used in patients with seizure disorders, prolonged QRS interval, or tolerance to benzodiazepines, including dependence on benzodiazepines and their derivatives [98].

Flumazenil is also used as an antisedation agent for benzodiazepines used to calm the patient during certain medical procedures. It also influences the restoration of proper respiratory and cardiovascular function by eliminating the central effect of benzodiazepine derivatives [103,107]. There are ongoing studies that indicate flumazenil as a potentially effective agent in the treatment of central disorders of excessive hypersomnolence resistant to treatment with available drugs that stimulate wakefulness [108] and studies on the use of flumazenil in the treatment of liver encephalopathy [109].

## 11. Glucagon

Glucagon is a white or faintly coloured crystalline powder, practically odourless and tasteless, practically insoluble in water. Isoelectric point of glucagon is 7.1 [110]. The primary structure of glucagon in humans is known as *NH_2_-His-Ser-Gln-Gly-Thr-Phe-Thr-Ser-Asp-Tyr-Ser-Lys-Tyr-Leu-Asp-Ser-Arg-Arg-Ala-Gln-Asp-Phe-Val-Gln-Trp-Leu-Met-Asn-Thr-COOH*. It is secreted mainly by the α cells from the pancreatic islets (the islets of Langerhans), located in the endocrine part of the pancreas, and released in response to hypoglycaemia, prolonged fasting, exercise, or protein-rich meals as the main hyperglycaemic hormone and is the counterbalancing hormone to insulin [111]. The precursor—proglucagon—whose expression was confirmed not only in α cells, but also in intestinal enteroendocrine L cells and, to a minor extent, in neurones of the brain stem and hypothalamus, is processed by processing enzymes prohormone convertase 1/3 (PC1/3) and prohormone convertase 2 (PC2). PC2 is responsible for processing proglucagon into glucagon in the pancreas, while PC1/3 analogously leads to the formation of glucagon-like peptide 1 (GLP-1) and glucagon-like peptide 2 (GLP-2) in the intestine and brain [112].

**Figure 5:**
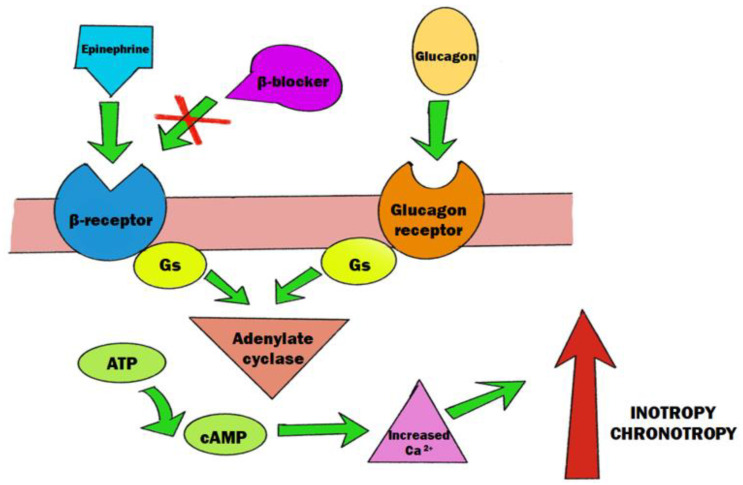
Glucagon exerts its inotropic and chronotropic cardiac effects by activating adenylate cyclase and increasing cAMP levels independently, bypassing β-receptors.

Cardiac glucagon receptors are coupled to Gs proteins—similar to β-receptors in the myocardium. When binding to them, glucagon activates adenylate cyclase, leading to an increase in cyclic adenosine monophosphate (cAMP) and the opening of L-type calcium channels (Figure 5). Intracellular calcium influx causes a positive inotropic and chronotropic effect, providing the same result as through β-adrenoreceptor stimulation by catecholamine-induced adrenoreceptor stimulation. Furthermore, enzymes within myocardial cells metabolise glucagon into its COOH-terminal fragment, called miniglucagon. Miniglucagon activates phospholipase A2 and stimulates arachidonic acid production, the accumulation of which in myocardial cells leads to increased cardiac inotropy [113].

In clinical use, glucagon appears in the form of dehydrated powder, termed “Glucagon Emergency Kit”, reconstituted with supplied sterile water to be administered intravenously, intramuscularly or subcutaneously, or as a purpose-formulated intranasal spray [114].

The use of high-dose glucagon in the treatment of β-blocker overdose can be found in various case reports dating back to the 1970s. According to some studies, haemodynamic parameters, such as median arterial blood pressure and median heart rate, increased after the administration of a higher dose of glucagon. However, glucagon therapy failure was also reported. The cause of such a diverse response to this treatment may lie in differences in the type and β-blocker ingested, and the specifics of the treatment [115]. The recommended loading dose in β-blocker poisoning is 5–10 mg in an adult patient and should be administered intravenously. Dosage can be reduced as patient condition continues to improve. Adverse effects include nausea or vomiting. Due to this, the sufficient amount of glucagon is often not available and dosing with other inotropic agents such as catecholamine infusions should be optimised first, making high-dose glucagon treatment a second-line treatment in β-blocker overdose [116,117].

Other than in β-blocker and calcium channel blocker overdose, glucagon is used mainly in the treatment of severe hypoglycaemia. Due to its simplicity of use and safe administration, glucagon is willingly used in the diabetic population, where obtaining intravenous access to provide glucose can be difficult and oral carbohydrate consumption can be dangerous, due to decreased levels of consciousness. Moreover, it induces hypotonia of the upper gastrointestinal and intestine tract—because glucagon is not only considered a diagnostic aid to imagine the gastrointestinal tract but is also used in abdominal vascular procedures, such as the treatment of oesophageal varices, to decrease peristalsis [114].

## 12. Hydroxocobalamin

Hydroxocobalamin is a form of manufactured injectable vitamin B_12_ [118]. It is a dark red solid with an odourless or faint odour of acetone (the melting point is about 200 °C). This substance is moderately soluble in lower aliphatic alcohols and practically insoluble in acetone, ether, petroleum ether, halogenated hydrocarbons, benzene, chloroform [119]. It is used in the treatment of vitamin B12 deficiency, including malignant anaemia, in addition to the treatment of cyanide poisoning, Leber optic nerve atrophy, and toxic optic neuritis [120].

Hydroxocobalamin is a synthetic form of vitamin B_12_ and a precursor to methylcobalamin; it plays a significant role in the development of the nervous system and in the metabolism of folic acid and adenosylcobalamin; it is involved in the metabolism of carbohydrates, amino acids, and fatty acids, and in the formation of myelin, the active forms of vitamin B_12_ [121]. Hydroxocobalamin affects the metabolism of methionine, folic, and malonic acids. Hydroxocobalamin is an antidote to cyanide poisoning. Cyanide binds to cytochrome C oxidase, which is the final complex in the electron transport chain. This process leads to inhibition of ATP production and cellular oxygen utilisation, preventing cellular respiration. Hydroxocobalamin contains cobalt compounds that can bind to cyanide to form cyanocobalamin. The kidneys excrete cyanocobalamin in urine. Hydroxocobalamin binds to transcobalamins, proteins that transport hydroxycobalamin to tissues. Cobalamin is absorbed in the ileum, stored in the liver, and excreted in the bile. Hydroxybalamin is used for patients who have suffered from fires most often in enclosed structures [122].

In cyanide poisoning, hydroxybalamin is used parenterally in intravenous injections under the trade name “Cyanokit”. The dose is based on how the patient responds clinically and the severity of their cyanide poisoning. For adults, the maximum administered dose is 10 g [123].

Hydroxybalamin also has other uses. It can be used to treat anaemia caused by vitamin B_12_ deficiency, which can be accompanied by subacute combined medullary degeneration. It is then administered parenterally. It also prevents anaemia. It can be used as a dietary supplement [122].

## 13. Insulin, Glucose (HIET)

**Figure 6:**
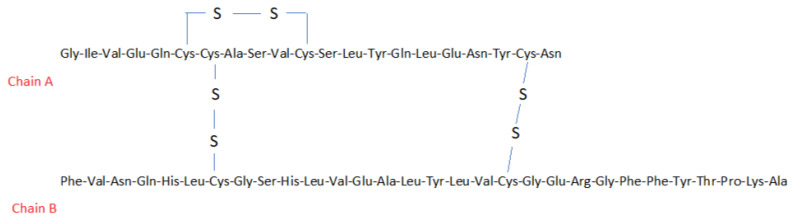
Chemical structure of insulin.

Insulin is a hormone produced by beta cells of the pancreas. It belongs to peptide hormones and is formed by combining 51 amino acid residues. It consists of two polypeptide chains (alpha and beta) and three disulphide bridges (chemical structure—Figure 6) [124]. Molecular weight of insulin is 5808 g/mol and its melting temperature is 81 °C [125].

Insulin has been used to treat CCB overdose as part of HIET therapy [126]. Calcium channel blockers (CCBs) are substances used to lower blood pressure, treat angina, or cardiac dysrhythmia. However, their overdose can cause headaches, flushing, tachycardia, and oedema. The toxic effects of CCBs are mainly due to conduction blockade and decreased contractility of heart muscle cells. Calcium channel blockers also block L-type calcium channels in peripheral vessels. This leads to shock due to a significant drop in blood pressure (BP) caused by excessive vasodilation [127]. Blocking of L-type calcium channels in pancreatic β-cells may contribute to decreased insulin secretion. Due to hypoinsulinaemia and insulin resistance from CCB overdose, cardiac muscle cells cannot freely use glucose for energy. This further reduces the contractility of the heart, leading to shock. Administering large doses of insulin as an antidote allows heart muscle cells to take up glucose and efficiently use it as an energy source. Insulin also has a direct concentration-dependent inotropic effect on human myocardial cells [127,128].

Hyperinsulinaemic euglycaemic therapy reduces vascular resistance (SVR), which can be beneficial in cases of intoxication. Reduced SVR results in a lower opposing force that the weakened myocardium must overcome to allow for adequate cardiac output and tissue perfusion [128].

High doses of insulin are used in HIET. Successful cases of use of HIET include 1 unit/kg intravenously. Despite the high doses of insulin used in HIET, numerous scientific studies suggest that HIET is effective and safe for patients [129].

## 14. L-Carnitine

L-Carnitine(3-hydroxy-4-trimethylamino-butyric acid) is an amino acid derivative and carnitine analogue. Physically, is a white, crystalline, hygroscopic powder (melting point of 197 °C). It is readily soluble in water and hot ethanol (solubility 2500 mg/mL), practically insoluble in acetone, ether, and benzene [130]. Levocarnitine is an important nutrient; a lot of it comes from the diet. It is also endogenously biosynthesised from dietary amino acids in the liver and kidneys. Most bodily carnitine is stored in skeletal muscles and other tissues with high energy demands. The total plasma concentration (free carnitine + acylcarnitine) is less than 0.6% of the total body stores. The main function of L-carnitine is to facilitate the transport of long-chain fatty acids from the cytosol to the mitochondria, where they undergo β-oxidation and produce acetyl-CoA [131].

**Figure 7:**
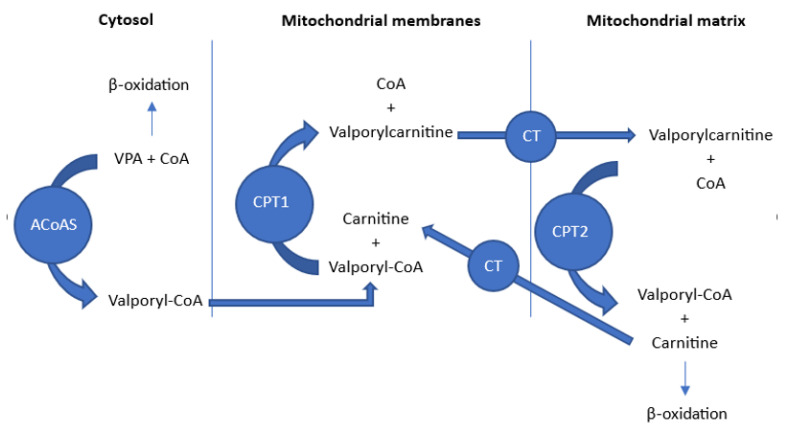
“L-carnitine shuttle” (based on [131]). VPA is activated in the cytosol, forming valproyl-CoA with reduced acetyl-CoA-SH. It then crosses the outer mitochondrial membrane as valproylcarnitine. Inside the mitochondrial matrix, valproylcarnitine is converted back to valproyl-CoA by PCT2 for slow β-oxidation. Carnitine prevents valproyl-CoA accumulation.

L-carnitine deficiency is an unusual problem in the healthy and well-nourished adult population, but several drugs, especially valproic acid (VPA), are associated with true carnitine deficiency [132]. VPA is a broad-spectrum antiepileptic drug. It is also used for several other neurological and psychiatric indications and is usually well tolerated, but serious complications, including hepatotoxicity, may occur. These complications may also arise after acute overdose of VPA [131]. As a branched chain carboxylic acid, VPA is extensively metabolised by the liver. Mitochondrial β-oxidation of VPA involves its transport within the mitochondrial matrix, using the same pathway as long-chain fatty acids [133]. This pathway consists of several steps and is sometimes called the “carnitine shuttle” (Figure 7) [131]. Carnitine is an essential cofactor in the beta-oxidation of fatty acids [134]. VPA stunts carnitine biosynthesis by decreasing the concentration of alpha-ketoglutarate and may contribute to carnitine deficiency [135]. Valproic acid-produced hepatotoxicity and hyperammonaemic encephalopathy can be led either by a pre-existing carnitine or by strictly VPA-induced deficiency. L-carnitine is used as an antidote in patients with valproic acid overdose hepatotoxicity who exhibit a reduced level of consciousness [131]. A single intravenous loading dose of 100 mg/kg is recommended, followed by infusions of maximum 3 g per dose every 8 h. Treatment should continue until ammonia levels decrease and the patient’s clinical condition improves or until adverse events related to L-carnitine occur [136].

Oral and intravenous L-carnitine is used for the treatment of primary and secondary carnitine deficiency due to inborn defects of metabolism. Intravenous L-carnitine is also used in dialysis patients with end-stage renal disease. L-carnitine administration in combination therapy improves glycaemic and insulin parameters in patients with type 2 diabetes. Improvements in some lipid parameters and diabetic peripheral neuropathy have been shown in diabetic patients whose diet is supplemented with L-carnitine [131].

## 15. Methylene Blue

Methylene blue is a dark green crystal or powder with a brown sheen (melting point is about 100–110 °C). The solubility in water, at 25 °C, is 43,600 mg/L. It also dissolves well in ethanol, chloroform, and weakly in pyridine. It is completely insoluble in ethyl ether. An aqueous or alcoholic solution takes on a deep blue colour [137].

**Figure 8:**
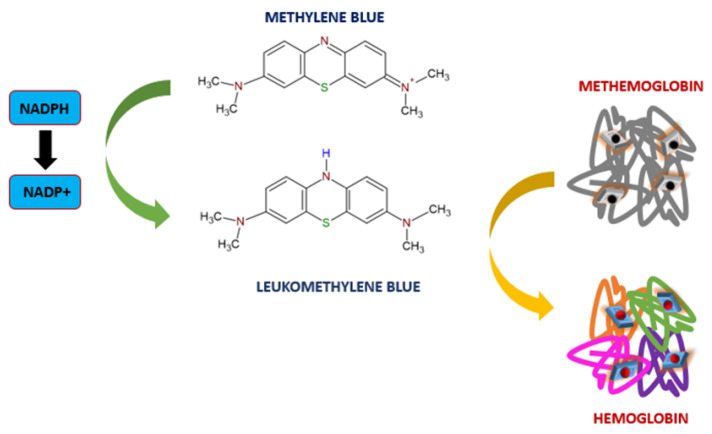
Mechanism of action of methylene blue. Methylene blue, in the presence of NADPH, converts to leucomethylene blue by obtaining electrons from NADH via complex I in the electron transport chain (ETC) within the inner mitochondrial membrane. Leucomethylene blue reduces methaemoglobin to haemoglobin.

Methylene blue in the presence of nicotinamide adenine dinucleotide phosphate (NADPH) is converted to leucomethylene blue. Methylene blue obtains electrons from NADH via complex I (NADH:ubiquinone oxidoreductase)—that is, one of the four complexes comprising the electron transport chain (ETC) located in the inner mitochondrial membrane. Leucomethylene blue reduces methaemoglobin to haemoglobin (Figure 8) [138].

In addition to the main indication, methylene blue should also be considered for ifosfamide-induced encephalopathy. A review of clinical cases indicates that patients who received methylene blue showed significant neurological improvement. Another indication in which methylene blue should be considered is catecholamine-resistant shock. Methylene blue has been described as increasing blood pressure by preventing vasodilation. It inhibits nitric oxide synthesis and also prevents guanylyl cyclase activation [139].

Its active form is methylthioninium chloride [137]. Intravenous methylene is used for the treatment of metheamoglobinemia in paediatric and adult patients. Metheamoglobinemia is a condition in which haemoglobin’s ability to transport oxygen is reduced by oxidation of the Fe^2+^ to the Fe^3+^. In both children and adults with metheamoglobinemia, the dose of methylene blue administered intravenously is 1 mg/kg bw of a 1% solution [140]. If clinical symptoms do not resolve, then this dose can be repeated. The maximum dose regardless of body weight is 100 mg [139].

In the future, methylene blue is also expected to be used as a safe photosensitiser in photodynamic therapy for the treatment of cancer and infectious diseases. In vivo and in vitro studies have yielded good results, but technical issues remain an obstacle, as it is difficult to create nanoparticles or microparticles infused with methylene blue [141]. Methylene blue, because of its ease of penetration into the blood–brain barrier and its antioxidant properties, is also a potential candidate for the treatment of neurodegenerative diseases. However, at this point, the results of clinical trials in mouse models are still controversial [138].

## 16. N-Acetylcysteine

N-acetylcysteine is a white crystalline powder with a slight acetic odour and a characteristic sour taste. It is soluble in water, alcohol, hot isopropyl alcohol, methyl acetate, and ethyl acetate. This substance remains stable when exposed to regular lighting conditions. It can withstand temperatures as high as 120 °C without undergoing any significant changes. Furthermore, it is considered non-hygroscopic. However, it should be noted that this substance is susceptible to oxidation when exposed to moist air [142]. *N*-acetylcysteine (NAC) was introduced to the market on September 14, 1963, in America, after FDA approval [143].

This compound is a derivative of the cysteine amino acid, a precursor to glutathione, which in the body plays a role in the detoxication of paracetamol [144]. For this reason, NAC is used as an antidote for paracetamol poisoning. In the event of an overdose, paracetamol is initially removed by conjugation with glucuronic acid and sulfuric acid. After using these metabolic possibilities, paracetamol is transformed in the liver by cytochrome P450 isoenzymes (mainly CYP2E1 and CYP3A4) into oxidative and highly hepatotoxic hydroxyl derivatives (N-acetyl-4-benzoquinimine-NAPQI). This compound is neutralised by a nonenzymatic reaction with sulfhydryl glutathione groups or with other compounds containing a thiol group, including N-acetylcysteine. In paracetamol overdose, the glutathione reserves in the body are used up, and the hepatotoxic metabolite NAPQI causes liver damage. Reconstruction of glutathione reserves depends on the amount of cysteine available. Here begins the role of NAC as an antidote, which in this case is converted into cysteine, which enables the synthesis of important glutathione [145].

If paracetamol poisoning is suspected, N-acetylcysteine should be administered immediately, within 4–8 h, not later than 14 h. The drug is administered intravenously (i.v.), or when the patient has not taken oral activated charcoal before [146].

The second application of N-acetylcysteine, right after the antidote in paracetamol poisoning, is its mucolytic effect, used, among others, in cystic fibrosis and chronic obstructive pulmonary disease. The mucolytic properties of NAC result from its ability to break disulphide bridges in the high molecular weight glycoproteins of which mucus is composed, thus reducing its viscosity and facilitating the expectation of secretions. In light of the latest research, NAC also finds much wider application, which results from its ability to restore glutathione reserves, which are becoming smaller in an increasingly ageing society. Currently, there is talk of the use of NAC in the aforementioned diseases of the respiratory system, but also in diseases of the liver and intestines, in metabolic diseases including nonalcoholic fatty liver disease, diabetes and polycystic ovary, in hypertension, in increasing fertility in men, in some cases of cancer, infectious diseases, neurodegenerative diseases, psychiatric diseases, and many others. Despite its simple structure, this compound offers us many therapeutic possibilities, of which the use has not yet been fully discovered [147].

NAC can also be used in the prevention and therapy of COVID-19 and influenza. Elderly people and individuals who have come into contact with carriers of SARS-CoV-2 can be targets for oral administration of NAC. A 600 mg dose twice a day is considered to reduce the risk of developing COVID-19, influenza, and influenza-like diseases [148].

NAC is the inhibitor NF-κB pathway, which is needed to replicate RNA viruses like human coronaviruses and influenza viruses. In addition, NAC reduces the production of proinflammatory interleukins, which leads to reducing monocyte migration [149,150].

Another potential target is a direct inhibitor of main protease (Mpro). Mpro plays a key role in viral replication. NAC, by binding to Cys145, an active site of protease, prevents virus replication [151].

Reduced glutathione (GSH) serves as a key defence mechanism. During COVID-19, the recycling of GSH increases, but it cannot keep up with the high demand caused by lung disease. This situation requires the synthesis of new GSH, whose rate-limiting substrate is L-cysteine. NAC penetrates cells where it is deacetylated to L-cysteine, increasing synthesis of GSH. Furthermore, NAC increases extracellular levels of L-cysteine by chain redox reactions in plasma. Extracellular L-cysteine is transported through transport channels into cells [148,152].

Intravenous administration of NAC can reduce mortality from COVID-19 by improving lung functions. SARS-CoV-2 causes a “cytokine storm”, which has a strong correlation with mortality. Neutrophiles produce reactive oxygen species (ROS) that can directly harm the lungs or convert into more damaging oxidant species such as OH-. Furthermore, ROS can amplify inflammation by activating NF-kB and upregulating the expression of various genes, including IL-6, TNF-α, and chemokines. NAC is an OH- scavenger that prevents cytokine storms and ROS-induced pulmonary oedema. Intravenous administration of NAC (40 mg/kg bw/day) for 3 days in patients with mild to moderate acute lung injury improved systemic oxygenation, reduced the need for ventilatory support, and slightly decreased the mortality rate. Higher doses are suggested to lead to better clinical outcomes [151].

## 17. Naloxone

Naloxone (*N*-allylnoroxymorphone) is an opioid receptor antagonist that is used in therapeutics to deescalate the adverse effects of opioids [153]. Naloxone (as hydrochloride) has the physical form of white powder. It is hygroscopic and soluble in water and 96% alcohol. As a drug, naloxone hydrochloride dihydrate is used. It was developed in the 1960s by the company Sankyo. This compound is a derivative of oxymorphone, where the N-methyl group has been replaced by an N-allyl group. The terminal half-life of this compound is 30 to 90 min, while oral bioavailability is very low due to high first-pass metabolism. The time to effect after intravenous administration is 1–2 min (onset), 5–10 (peak), and it is metabolised mainly by the liver [154]. The drug is highly lipophilic and, therefore, reaches high concentrations in the brain where its target site of action is located [155].

**Figure 9:**
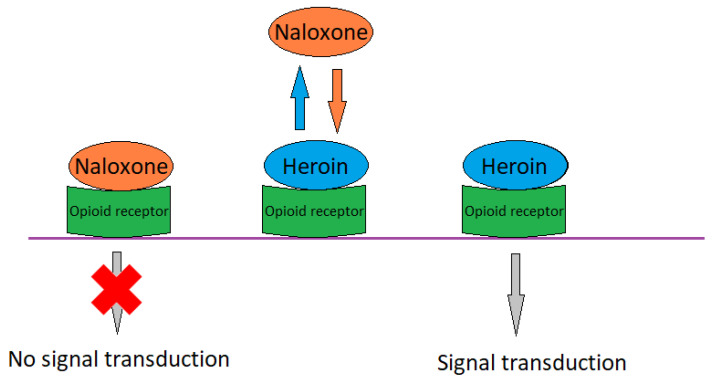
Mechanism of action naloxone (based on Straus, M. et al. [156]). Naloxone binds to the mu-receptor but does not activate the receptor response like an agonist. Additionally, it blocks the binding of an agonist that would activate the receptor. This makes naloxone an effective antidote for opioid overdose due to its pharmacological properties.

Naloxone works by competitively binding to their respective target receptors. The substance is a competitive antagonist of μ, δ, and κ receptors, thus antagonising the action of endogenous (e.g., endorphin) as well as exogenous ligands (e.g., morphine, fentanyl). Naloxone has a higher affinity for the μ receptor than for the δ and κ receptors and competes for the active receptor site with heroin and morphine, generally displacing administered substances and eliminating their undesirable effects, such as respiratory depression (Figure 9) [154]. In addition to the emergency treatment of opioid overdose, naloxone has also used in addiction therapy, in combination with buprenorphine, to discourage drug use. When administered orally, it acts on opioid receptors in the enteric nervous system, alleviating symptoms such as postoperative paresis and constipation following the use of oral opioids [79].

There is currently an epidemic of opioid poisoning, particularly in the United States. It was caused primarily by the misuse of prescription opioids and increased availability, so naloxone as an opioid receptor antagonist is an important and effective antidote that has saved many lives. It was first introduced to the market in 1971 as a prescription drug in the United States and a year later internationally [157]. Naloxone is most commonly administered intravenously but can also be administered intramuscularly subcutaneously or by intraosseous line. The patient needs to be closely monitored after taking the preparation, so medical help must be notified even if the patient’s condition has improved, because a repeat dose may be needed. The side effects of naloxone mainly consist of rapid opioid withdrawal, associated with symptoms such as nausea, vomiting, aggression, diarrhoea, and anxiety. Therefore, it remains important to select an appropriate dose that will compensate for the effects of opioids without causing withdrawal symptoms [158].

## 18. Octreotide

Octreotide is a peptide with the formula H-D-Phe-Cys(1)-Phe-D-Trp-Lys-Thr-Cys(1)-Thr-ol. At room temperature, it is a solid with a melting point of 153–156 °C [159].

Octreotide works by binding to somatostatin receptors. It inhibits the cellular production of cyclic adenosine monophosphate and the influx of calcium through the voltage-gated calcium channel, resulting in hyperpolarisation of pancreatic cells and reduced insulin secretion. This effect is mediated by the SSTR2 subtype and, to a lesser extent, by SSTR3 and SSTR5, G protein-coupled receptors [160].

The octreotide in the beta cells of the pancreatic islets prevents the influx of calcium, which inhibits the release of insulin. Octreotide disrupts the stimulus promoted by sulfonylureas, preventing the process of KATP channel closure, calcium influx, and insulin release. It is used as an antidote to poisoning with oral hypoglycaemic drugs with derivatives of sulfonylurea [161]. The use of octreotide alone or in combination with standard dextrose and oral carbohydrate therapy through multiple studies has been supported by evidence [162].

The octreotide is well tolerated by the body and is effective in reducing sulfonylurea-induced hypoglycaemia. Table 1 shows the use of octreotide as an antidote in poisoning with hypoglycaemic drugs with sulfonylurea derivatives. After the last dose of octreotide, blood glucose should be monitored approximately every 15–60 min for up to 8 h to exclude prolonged hypoglycaemia [163].

Octreotide is also used in the treatment of acromegaly. It has brought tremendous benefits to most patients: marked relief from symptoms occurs quickly after starting therapy. Disease-related comorbidities, including cardiac and respiratory disorders, improve with long-term therapy. Furthermore, most TSH-secreting pituitary adenomas express SST2 and respond to octreotide by inhibiting hormone release and controlling tumour growth and overall clinical improvement. Octreotide is also used in the treatment of neuroendocrine tumours of the stomach, intestines, and pancreas. It helps control diarrhoea and redness attacks, relieves dehydration, hypokalaemic alkalosis, peptic ulcers, hypoglycaemic attacks, and necrolytic skin lesions. The direct clinical effects of somatostatin analogue therapy on quality of life in most cases brings about a significant improvement in health and general wellbeing [165].

## 19. Oxygen

Oxygen is a chemical element denoted by the symbol O. Its atomic number is 8. It has been classified as a nonmetal. At room temperature, oxygen is a gas. This name comes from Greek words that mean *oxys* for “acid” and *genes* for “forming”, because French chemist Antoine-Laurent Lavoisier thought oxygen was integral to all acids. Initially, scientists called it “dephlogisticated air” and “fire air” in their experiments. It is the third most common element in the universe; it makes up almost 21% of the Earth’s atmosphere. Oxygen accounts for almost half of the mass of the Earth’s crust, two thirds of the mass of the human body, and nine tenths of the mass of water. A large amount of oxygen can be obtained by fractional distillation of liquefied air and by electrolysis of water or heating of potassium chlorate (KClO_3_) [166]. Oxygen is an odourless, colourless, and tasteless gas. It exists in three allotropic forms as normal oxygen, diatomic oxygen, and triatomic oxygen (ozone). Allotropes differ in their physical and chemical properties. Oxygen supports combustion and reacts with many compounds [167].

The best antidote for carbon dioxide poisoning is oxygen. The indications of carbon monoxide (CO) intoxication are nonspecific; each time they are different, from dizziness and headache to more serious effects, such as loss of consciousness and even death. They depend on concentration and exposure time. When carbon monoxide poisoning occurs, the oxygen-carrying capacity of the blood is reduced, the cellular respiratory chain is impaired, and tissue in the heart muscle and brain can be damaged by immunomodulatory processes. People affected by this kind of poisoning began to receive oxygen; the faster it worked, the greater the results achieved were. The higher the partial oxygen pressure (pO_2_), the faster the CO was eliminated. The elimination half-life of CO after breathing room air is approximately 320 min and can be shortened to 74 ± 25 min by administering 100% oxygen to patients. Treatment with hyperbaric oxygen (pO_2_ = 2.5 bar) reduced the half-life to approximately 20 min. In animals, hyperbaric oxygen has also been shown to reduce inflammatory processes, mitochondrial dysfunction, and lipid peroxidation [168].

Oxygen is an essential element needed for human survival. Medical oxygen therapy is used most frequently to lower blood oxygen levels and has the secondary effect of reducing resistance to blood flow in the affected lung, which reduces the burden on the cardiovascular system to oxygenate the lungs. Oxygen therapy is used to heal the lungs, to treat emphysema, any disease that impairs the body’s ability to take in and use oxygen gas, and to increase pressure in the pulmonary artery. Oxygen therapy is very effective; it even improves the efficiency of oxygenation of cells at a low rate of tissue perfusion. Hypoxic ventilation is driven by oxygen molecules acting on the chemoreceptors in the carotid arteries, which relay sensory information to higher processing centres in the brainstem. It also reduces hypoxia-induced mitochondrial depolarisation that generates reactive oxygen species and/or apoptosis. Studies on hyperbaric oxygen therapy performed in neonatal rats have shown that oxygen supplementation can induce neural stem cell proliferation, positively affecting neurological recovery after injuries [169].

Oxygen therapy improves gas exchange and oxygen delivery to tissues by increasing arterial oxygen pressure. Oxygen plays a key role as an electron acceptor during oxidative phosphorylation in the electron transport chain by activating cytochrome c oxidase (the end enzyme of the electron transport chain). This process allows for aerobic respiration in organisms, where ATP molecules are produced and used as an energy source in many tissues. Cellular activity at the mitochondrial level is restored by adequate oxygen administration and metabolic acidosis is reduced [169].

## 20. Physostigmine

It is an organic chemical compound, an alkaloid, and an acetylcholinesterase inhibitor. It is obtained from ordeal bean (*Physostigma venenosum*). The exact systematic name of physostigmine is methylcarbamate-(3a S-cis)-1,2,3,3a, 8,8a-hexahydro-1,3a, 8-trimethylpyrrolo [2,3,b]indole-5-yl [170]. After crystallisation from diethyl ether or benzene, it takes the form of orthorhombic columns or groups of leaf-shaped crystals. Its melting temperature is 105 to 106°C. It is slightly soluble in water, and better in alcohol, benzene, chloroform, and oils. Physostigmine also occurs in the form of a series of salts, the most common of which is physostigmine salicylate, which forms needle-shaped crystals with a melting point of 185–187 °C [171].

In the form of sulphate, it is present as hygroscopic scales with a melting point higher than other varieties, equal to 140 °C (after drying 100 °C) and with good solubility in water and ethanol, and very low in diethyl ether [172].

This alkaloid is an inhibitor of acetylcholinesterase in peripheral and central cholinergic structures. Its administration causes an indirect increase in the stimulation of parasympathetic endings by increasing the concentration of acetylcholine in the presynaptic spaces. The effect of physostigmine can be compared to that of another alkaloid, pilocarpine, but physostigmine is used much less frequently. Both of these alkaloids cause a drop in intraocular pressure, increase intestinal peristalsis, and cause contractions of the smooth muscles of some organs, such as the bladder [173].

The indication for the use of physostigmine is the treatment of severe anticholinergic toxicity. The dosage during this situation is 1–2 mg i.v. over 5 min, if necessary, every 5 min additional doses of 0.5–1 mg are administered until cholinergic signs, or delirium, resolve [174].

Physostigmine is also used in the treatment of glaucoma; it is the drug of choice because it causes a drop in intraocular pressure and narrows the pupils [175].

## 21. Prussian Blue

The ferrocyanide oxidation reaction leads to the formation of a dark blue pigment, which is Prussian blue (PB). Interestingly, the first synthetically created polymer with a coordinating function was considered the substance mentioned above [176]. The construction of this compound is based on the crystal lattice with a cubic structure, which includes Fe (III) and Fe (II) ions. In terms of physical properties, Prussian blue is characterised by the ability to form colloids and may or may not dissolve in water. Heavy metal cations and water molecules can fit into the PB structure, which is due to the appropriate width of the channel diameter (3.2 A) [177].

As a consequence, thallium and caesium particles, which are toxic to the human body, are sequestered under the influence of Prussian blue. The biological half-life of these metals in the gastrointestinal tract is shortened, which is due to oral ingestion of PB to facilitate the excretion of these radioisotopes from the body. For example, caesium is bound to Prussian blue through a series of processes, including physical adsorption, ion trapping, hydronium cation exchange, and potassium ion exchange [178]. Faeces are the form in which the compound is excreted, which was found to represent 99% of the dose [179]. As for the doses recommended in case of poisoning, for thallium it is 20 g/day, and for caesium it is 3 g/day [178,180].

Prussian blue and its nanoparticles in particular are also used in the removal of ROS. In a mouse, the property of PB nanoparticles may contribute to the annihilation of inflammatory bowel disease [181]. PY (Prussian yellow) and BG (Berlin green) are the two forms into which blue transforms through its ability to donate electrons. Pluricoloured blue also acts as a wound healing agent; this study was carried out in mice. As a consequence, adequate wound healing was achieved, which is the result of the fact that processes such as neovascularisation and angiogenesis were induced by the use of the PB nanozyme [182].

## 22. Protamine Sulphate

Protamine (protamine sulphate) is a highly basic, polycationic, low molecular weight protein with a molecular weight of 5000. Today, protamine is produced using recombinant technology, but was originally isolated from salmon sperm [183]. Discovered in salmon sperm heads in the late nineteenth century and introduced in 1939, protamine sulphate still occupies an important therapeutic role today as perhaps the only viable option to reverse the anticoagulant effect of heparin [184].

It is used to reverse heparin-induced anticoagulation after separation from the extracorporeal circulation, reducing the risk of postoperative bleeding or reversing the anticoagulant effect of unfractionated heparin during dialysis, invasive vascular procedures, and acute ischaemic strokes [185].

Unfractionated heparin is a strongly anionic anticoagulant that forms a salt aggregate (heparin/protamine complex) when it comes into contact with the positive cationic arginine peptide in protamine [183]. Protamine sulphate is an antagonist of heparin and inhibits heparin-induced anticoagulation by forming an inactive and stable heparin/protamine complex without anticoagulant properties [186]. Protamine has a rapid onset of action, neutralising unfractionated heparin in 5 min, and has a relatively short half-life of about 10 min—in healthy patients with no heparin present in the body, it was determined to be approximately 7.4 min, while in surgical patients undergoing cardiopulmonary bypass surgery with the use/presence of heparin in the body, the recorded half-life was approximately 5 min [183,187]. The metabolism of the inactive heparin–protamine complex has not yet been thoroughly elucidated. Some studies point to its metabolism in the liver, while others point to the kidneys as the site of metabolism and excretion of the complex [183,188].

Protamine sulphate, unbound to heparin, has a weak anticoagulant effect due to its interaction with platelets, fibrinogen, and other blood proteins, but is much weaker than heparin. It also prolongs the euglycaemic phase, as it is used as an excipient in some injectable insulin preparations. Protamine can be used to partially reverse the effects of low molecular weight heparins, including enoxaparin, dalteparin, and tinzaparin, but according to studies, protamine does not completely neutralise the effects of low molecular weight heparins [186]. This use has not been approved by the FDA [183].

In a surgical setting, protamine is usually administered intravenously, most often via a peripheral intravenous line as a slow infusion lasting 10 to 15 min, aimed at reducing the probability of adverse effects such as anaphylactic reactions that can occur when administered too quickly. The incidence of anaphylactic reactions ranges from 0.06% to 10.6%. There are also reports of liver and kidney tissue damage [183]. It is recommended to select the dose of protamine sulphate based on its titration with heparin in vitro, with simultaneous measurement of clotting generation time (ACT) [186]. Heparin neutralisation occurs within five minutes after intravenous administration of protamine sulphate [188]. The effectiveness of protamine to reverse the effect of heparin can be assessed by measuring activated clotting time or performing a thromboelastogram [183].

Protamine is a drug used to reverse the effects of unfractionated heparin and during heparin overdose [183,188].

Currently, there are numerous studies for other heparin antagonists and antidotes; however, most substitutes have similar and unacceptable side effects. Therefore, the current gold standards for anticoagulation and anticoagulation reversal are still heparin and protamine [184].

## 23. Pyridoxine

Pyridoxine is a white powder soluble in water with a melting point of 159–162 °C. It is one of the three natural forms of vitamin B_6_ (pyridoxine, pyridoxal, and pyridoxamine). All of them convert inside the human body into an active coenzyme, pyridoxal 5-phosphate, which is essential for many other enzymes, including serine-hydroxymethyltransferase, cystathionine-beta-synthase/lyase, and aromatic L-amino acid decarboxylase. Pyridoxine plays an important role in the proper functioning of the nervous system, because it allows for neurotransmitter and myelin synthesis and controls glutamate excitability, and neuronal metabolism of amino acids, DNA/RNA [189].

Pyridoxine as an antidote is used primarily to antagonise the effects of isoniazid (INH) toxicity or overdose [190]. Other toxicological uses include false morel poisoning (Gyromitra), exposure to hydrazine, and crimidin toxicity [191]. Isoniazid is a drug with a variety of adverse effects; its interference with vitamin B_6_ results in inducing peripheral neuropathy [192]. Isoniazid metabolites inactivate pyridoxine species and inhibit pyridoxine phosphokinase, an enzyme essential for the transformation of pyridoxine into pyridoxal 5-phosphate [190]. In this way, INH contributes to pyridoxine deficiency and consequently to reduced production of g γ-amino butyric acid (GABA). The described mechanism is the leading cause of seizures in cases of acute INH toxicity. Pyridoxine directly antagonises the effects of INH by binding to it, replenishing pyridoxine stores, and facilitating the production of GABA [98]. If the dose of used INH is known, it is administered gram for gram. If the amount of INH taken is not known, 5 g of pyridoxine is administered intravenously to adult patients [193].

Pyridoxine administered in lower doses (compared to those used to reverse the toxicity of INH) could help treat ethylene glycol toxicity, theophylline-induced seizures, or acute alcohol intoxication [193]. Some studies also suggest that pyridoxine might be useful for alleviating nausea and vomiting in early pregnancy [194].

## 24. Starch

**Figure 10:**
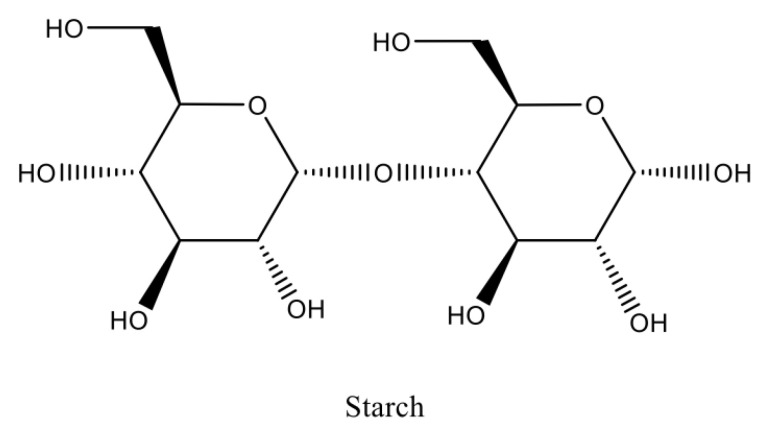
Chemical structure of starch.

**Figure 11:**
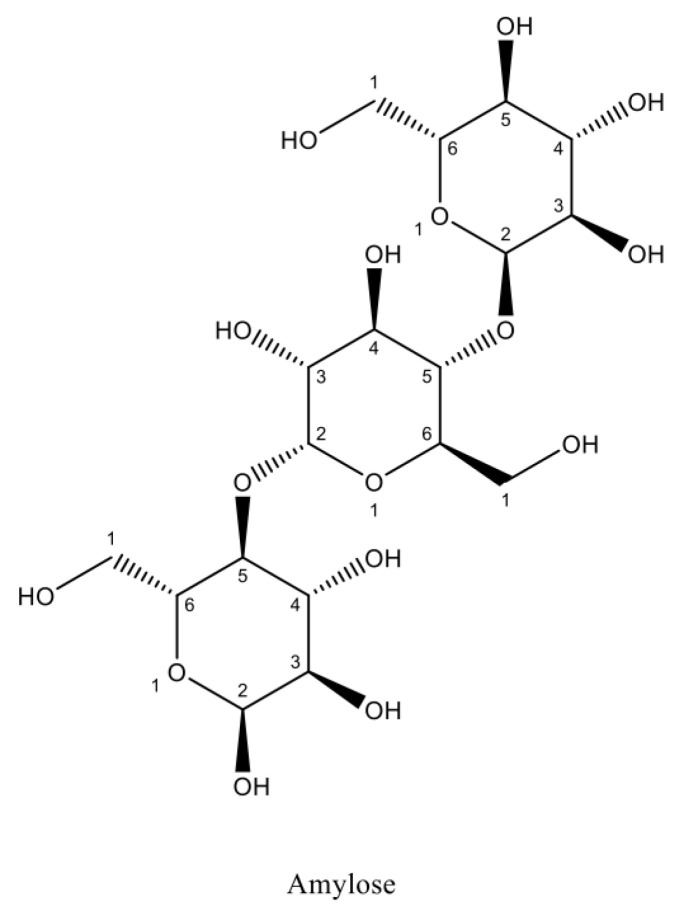
Chemical structure of amylose.

**Figure 12:**
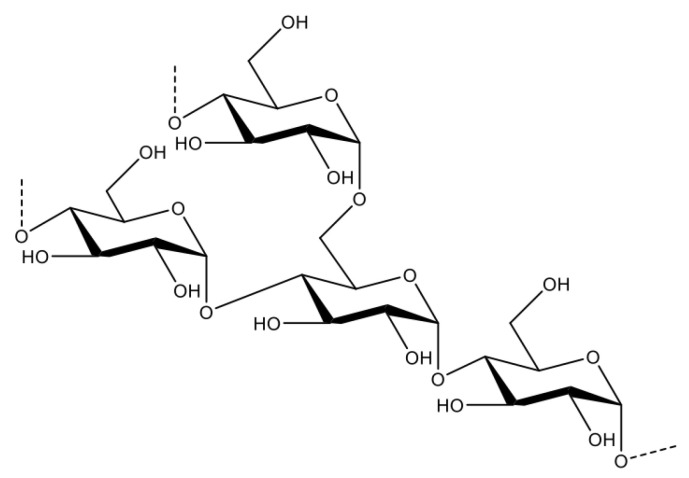
Chemical structure of amylopectin.

Starch (Figure 10) is used for energy storage in plants. It is found in all seeds and tubers of plants and is the form in which glucose is stored for later use. Starch can be separated into two main polysaccharides: amylose (Figure 11) and amylopectin (Figure 12). Although the starch from each plant is unique, most starches contain 20% to 25% amylose and 75% to 80% amylopectin. The complete hydrolysis of amylose and amylopectin yields only d-glucose. Amylose is composed of unbranched chains of up to 4000 d-glucose units joined by a-1,4-glycosidic bonds. Amylopectin contains chains up to 10,000 d-glucose units, also joined by a-1,4-glycosidic bonds. In addition, there is considerable branching from this linear network. At the branches, new chains of 24–30 units are started by a-1,6-glycosidic bonds [195]. Depending on the botanical origin and genetic background, starch has different chemical structures, such as branch chain lengths and phosphate derivatives, and different functional properties. Various physical, chemical, and enzymatic modifications change and improve the functional properties of starch and facilitate its utilisation for different purposes [196].

Starch is used for iodine poisoning and is administered as a starchy food (e.g., potato, flour, or corn starch) or milk to convert iodine to the less toxic iodide. Irrigation of the stomach with starch solution through a nasogastric tube can be used and will turn the gastric effluent dark blue-purple. This change in colour can be used as a guide to determine when lavage can be terminated [197,198].

A 15 g dose of starch solution dissolved in 500 mL of water can be administered orally or intranasally [199].

Using iodine to test for the presence of starch is a common experiment. A solution of iodine (I2) and potassium iodide (KI) in water has a light orange-brown colour. The colours are caused by so-called charge transfer (CT) complexes. Molecular iodine (I2) is not easily soluble in water, which is why potassium iodide is added. Together, they form polyiodide ions of type In–, for example, I3–, I5–, or I7–. The negatively charged iodide in these compounds acts as a charge donor, and the neutral iodine acts as a charge acceptor. Electrons in such charge-transfer complexes are easily excited to a higher energy level by light. The light is absorbed in the process, and its complementary colour is observed by the human eye. The exact structure of the polyiodides inside the amyloid helix is unclear. The amylose–iodine complex is amorphous (i.e., it does not form ordered crystals), which makes it difficult to determine its structure. The species within the helix has been proposed to be repeated units I3 or I5 [200].

## 25. Vitamin K

Vitamin K is a term used to describe many similar compounds that have a common physiological function. The same structure can be found in each of these compounds, the 2-methyl-1,4-naphthoquinone core, that is, menadione. Vitamin K_3_ is made only of this core. Other known forms are vitamin K1 (phytomenadione), which can be found in plant sources, and vitamin K_2_ (menaquinone), which can be found in animal sources. Vitamin K_1_ and vitamin K_2_ are natural vitamers, while vitamin K_3_ is a synthetic form that was used in a treatment for vitamin K deficiency. However, it is no longer used for this because it interferes with the function of glutathione. All forms of vitamin K are fat-soluble [201].

The main and most well-known function of vitamin K is its role in the coagulation process. It participates in γ-carboxylation of the gla domain in “Gla proteins”. It functions as a cofactor for γ-glutamylcarboxylase. This enzyme catalyses carboxylation of the amino acid glutamic acid (Glu) to γ-carboxyglutamic acid (Gla). Carboxylation is necessary for proteins to chelate calcium. Some of these proteins play a key role in the coagulation cascade: factors II, VII, IX, and X can be described as procoagulant, while proteins C, S, and Z are anticoagulant [202,203]. Vitamin K is used to treat rodenticide poisoning with “rat poison”. Rodenticides usually consist of anticoagulants, such as warfarin, coumarins, or indandiones. We can identify the poisoning by examination of the PT/INR. Coumarine or indandione poisoning results in an increase in this indicator. Prothrombin levels can also be reduced and occur in 24 to 48 h. In the event of poisoning, vitamin K_1_ is administered orally to protect against the anticoagulant effect if there is an increase in PT/INR. The IV route is restricted to patients who cannot take it using any other method of administration because, in that case, there is a risk of anaphylactoid reaction. Dosage for adults is 10–50 mg of vitamin K_1_ 2–4 times a day. If PT/INR decreases, the dose can also be decreased accordingly. Treatment may last even 3–4 months [204].

## 26. Conclusions

Poisoning remains a significant cause of injury-related death, with chemical weapons posing a threat to both military and civilian populations. Emergency medicine principles should be followed in cases of poisoning or overdose, including monitoring vital signs and providing supportive care. Antidotes are an important aspect of poisoning therapy, with a scientific approach dating back to the last century. While some antidotes have become redundant with modern intensive care, they still play a critical role in saving lives or reducing the severity and duration of toxicity. However, access to antidotes is a concern in developing countries without resuscitation and intensive care facilities. The development of new antidotes and poisoning therapies is a very important future direction. There is great potential for development of new supramolecular antidotes.

This review was possible thanks to the involvement of students who were part of the toxicological science club “Paracelsus” (Institute of Medical Studies, Medical College, Rzeszów University), who conducted a review as part of the project implementation “Toxicovigilance, poisoning prevention and first aid in poisoning with xenobiotics of current clinical importance in Poland”, grant number SKN/SP/570184/2023.

## Figures and Tables

**Table 1:** Specifics of octreotide as an antidote in poisoning hypoglycaemic drugs. (based on [164]).

Characteristic	Specific Parameter Value
Mechanism of action	The compound binds to somatostatin-2 receptors that are located on pancreatic β cells, and this prevents the influx of calcium that is required for insulin secretion.
Peak effect	Half hour
Preferred route	Subcutaneous
Dosing schedule—adults	50–100 μg every 6–12 h
Adverse effects	Hyperglycaemia, injection site pain, nausea, abdominal pain, flatulence, diarrhoea
